# Inhibition of bromodomain and extra‐terminal proteins increases sensitivity to venetoclax in chronic lymphocytic leukaemia

**DOI:** 10.1111/jcmm.14857

**Published:** 2019-12-10

**Authors:** Giovanna Carrà, Paolo Nicoli, Marcello Francesco Lingua, Beatrice Maffeo, Antonio Cartellà, Paola Circosta, Mara Brancaccio, Guido Parvis, Valentina Gaidano, Angelo Guerrasio, Giuseppe Saglio, Riccardo Taulli, Alessandro Morotti

**Affiliations:** ^1^ Dept. of Clinical and Biological Sciences University of Turin Orbassano Italy; ^2^ Dept. of Oncology University of Turin Orbassano Italy; ^3^ Dept. of Molecular Biotechnology and Health Sciences University of Turin Turin Italy; ^4^ Division of Hematology Ospedale Mauriziano Torino Italy

**Keywords:** BCL‐2, BET inhibitor, BRD4, chronic lymphocytic leukaemia, venetoclax

## Abstract

The development of drugs able to target BTK, PI3k‐delta and BCL2 has dramatically improved chronic lymphocytic leukaemia (CLL) therapies. However, drug resistance to these therapies has already been reported due to non‐recurrent changes in oncogenic pathways and genes expression signatures. In this study, we investigated the cooperative role of the BCL2 inhibitor venetoclax and the BRD4 inhibitor JQ1. In particular, we found that JQ1 shows additional activity with venetoclax, in CLL cell lines and in ex vivo isolated primary CD19^+^ lymphocytes, arguing in favour of combination strategies. Lastly, JQ1 is also effective in venetoclax‐resistant CLL cell lines. Together, our findings indicated that the BET inhibitor JQ1 could be a promising therapy in CLL, both as first‐line therapy in combination with venetoclax and as second‐line therapy, after the emergence of venetoclax‐resistant clones.

## 1 INTRODUCTION

The newest drugs to target chronic lymphocytic leukaemia (CLL) include the inhibitors of the intracellular B‐cell receptor signalling (BCR inhibitor)^1^ and the BCL2 inhibitor venetoclax.^2^, ^3^ B‐cell receptor signalling inhibitors incorporate the direct BTK inhibitor ibrutinib and the inhibitor of PI3K‐delta,^4^ a BTK downstream effector, idelalisib. Both ibrutinib and idelalisib have entered the clinical field with impressive results in chemotherapy‐refractory CLL patients. However, both drugs are less effective with p53 mutated/deleted CLL cells. This type of CLL remains highly challenging form which should better benefit from the treatment with the BCL2 inhibitor, venetoclax, which acts as a pro‐apoptotic trigger.^5^ With such a remarkable option of drugs and the possibility to target p53 mutated/deleted clones, CLL should be considered as an easily treatable cancer and the intent to eradicate the disease no longer a fleeting mirage. Unfortunately, cases of resistance to each of these novel drugs have already been reported and mechanisms of resistance deeply investigated.^2^, ^6^, ^7^, ^8^ Interestingly, however, no recurrent abnormalities or mutations have been associated with a specific pattern of resistance, posing some concerns on how resistant patients can be further treated.

It should be noted that in all the trials with these new drugs in the CLL context, idelalisib, ibrutinib and venetoclax are always used as single agents.^9^ In the dynamic process of clonal evolution of cancer, the administration of a single agents could simply represent a selective pressure to promote the evolution of sub‐clones, thereby selecting those with a resistant behaviour.

It is indeed mandatory to design combinatorial strategies to be administrated upfront the development of resistant clones or alternatively move into a step by step (drug by drug) process.

Venetoclax resistant clones originate from cells where a set of genes are differentially expressed when compared with parental clones. Among these, the major determinants are MCL‐1 and BCL‐X_L._ 10, 11 This means that, parallel to apoptosis promotion through BCL2 modulation, venetoclax affects the overall gene expression machinery. Some of these genes may favour cells to survive.

In line with these considerations, a perfect drug to associate with venetoclax would be a drug that transiently impairs gene expression during venetoclax administration, preventing the activation of compensatory mechanisms that inhibit or affect the induction of apoptosis.

The bromodomain and extra terminal BET family proteins comprise four members (BRD2, BRD3, BRD4 and the testis‐specific BRDT), which contain tandem bromodomains that allow to interact with acetylated lysines of histones and regulate gene transcription of relevant oncogenes, such as MYC, CDK genes, cyclin D1 and BCL2.^12^, ^13^, ^14^

Currently, various BET inhibitors have been developed and few of them are under investigation in clinical trials.^15^, ^16^ However, while BET inhibitors were consistently described as highly effective drugs in in vitro experiments, few in vivo observations and early clinical trial reports had already posed some concerns regarding the potential development of resistance to BET inhibitors, when administrated as single agents.^16^ In the case of AML, for instance, treatment with BET inhibitors promotes resistance through the up‐regulation of BET target genes.^17^

In this report, we have investigated the mechanisms of venetoclax resistance in CLL. In addition, we have demonstrated the efficacy of BET inhibitors, JQ1, in combination with venetoclax and against venetoclax resistant clone.

## 2 EXPERIMENTAL PROCEDURES

### 2.1 Cells and primary human samples

Human MEC‐1 cell line was gently provided by Prof. Deaglio (University of Turin)^18^, ^19^ and was grown in IMDM medium (Life Technologies) supplemented with 10% foetal bovine serum (FBS; Sigma Aldrich), 1% penicillin/streptomycin (EuroClone) and maintained at 37°C in a 5% CO_2_ humidified atmosphere. EHEB cell line was purchased from DSMZ and was maintained in RPMI medium 10% FBS and 1% penicillin/streptomycin (EuroClone). The lymphoid nature of this cell line was authenticated by flow cytometry (CD19^+^ positivity).

Human samples were collected from untreated CLL patients at the San Luigi Hospital (Orbassano, Italy), following informed consent and with obscured identity, as processed as previously described.^20^ Briefly, CLL cells were isolated from peripheral blood samples by density gradient centrifugation accordingly to Ficoll procedure (Sigma‐Aldrich). Following centrifugation at 400 *g* for 20 minutes, CD19+ lymphocytes were isolated accordingly to the Miltenyi Biotec protocol (Miltenyi Biotec, #130‐050‐301).

The project was reviewed and approved by the institutional ethical committee (code #10/2013).

### 2.2 Gene expression analysis

RNA was extracted from cells using TRIzol as described.^21^ 1 μg of total RNA was used for reverse transcription with iScript cDNA Synthesis Kit (Bio‐Rad) according to the manufacturer's protocol. Real‐time PCR was performed with iQ SYBR Green (Bio‐Rad) with the following primers:

- BRD2_for: 5′‐GGAAGATGAGGAGGACGAGG‐3′;
- BRD2_rev: 5′‐TGGGCTTGGATATTGGACCC‐3′;
- BRD4_for: 5′‐ATACCTGCTCAGAGTGGTGC‐3′;
- BRD4_rev: 5′‐TGTTCCCATATCCATAGGCGT‐3′;
- hHuPO FW: 5′‐GCTTCCTGGAGGGTGTCC‐3′;
- hHuPO RV: 5′‐GGACTCGTTTGTACCCGTTG‐3′

Real‐time PCR parameters were cycle 1, 95°C‐3 minutes; cycle 2, 95°C‐15 seconds, 60°C‐30 seconds for 40 cycles. The 2^−ΔΔCT^ method was used to analyse the data. hHuPO was used to normalize the results.

### 2.3 Cell proliferation assay, cell‐cycle analysis and assessment of apoptosis

Cells were plated in 96‐well plates at the density of 1.5 × 10^3^ cells/well. Proliferation was evaluated by CellTiter‐Glo (Promega) following the manufacturer's instructions. Cells were plated at a density of 2.5 × 10^5^ in 6‐well plates and then treated or not with JQ1 (0.5 μmol/L) for 2 days. After being harvested and washed with PBS, cells were treated with RNAse (0.25 mg/mL) and stained with propidium iodide (50 μg/mL). The cell‐cycle distribution in G0/G1, S and G2/M phase was calculated using the CellQuest program (BD Biosciences).

Apoptosis was measured by flow cytometry after staining with Annexin V. Briefly, after 2 days with or without JQ1 (0.5 μmol/L), venetoclax (0.5 μmol/L) or a combination of these two drugs. Cells were washed in PBS and incubated for 15 minutes at room temperature in HEPES buffer solution (10 mmol/L HEPES, pH 7.4, 140 mmol/L NaCl, 2.5 mmol/L CaCl2) with 2.5 μL Annexin V Fitc/PI (BD Biosciences). Cells were analysed by FACScan using CellQuest Software (BD Biosciences).

The combination index (CI) for drug combination was calculated with the available software CalcuSyn. CI values < 1.0 indicate a synergistic interaction of the two drugs in the combination.

### 2.4 Cell lysis and Western blot assay

Cells were lysed in lysis buffer containing 150 mmol/L NaCl, 1 mmol/L EDTA, 50 mmol/L Hepes (pH 7.5), 1% Triton X‐100 and 10% glycerol. Protein lysates were resolved in 4%‐15% SDS‐PAGE gels transferred into nitrocellulose filters. Proteins were visualized with peroxidase‐conjugated secondary antibodies and chemiluminescence reagent (BIORAD, #170‐5060).

### 2.5 Anchorage‐independent cell‐growth assay

Cells were suspended in 0.45% type VII low‐melting agarose in 10% IMDM at a density of 5 × 10^3^ cells/well and plated on a layer of 0.9% type VII low‐melting agarose in 10% IMDM in 6‐well plates then cultured at 37°C with 5% CO_2_. After 2 weeks, colonies were counted, and images were acquired at 5× magnification.

### 2.6 Antibodies and inhibitors

GAPDH (#5174), pERK1/2 (# 9101S), ERK1/2 (# 4695S), pAKT (# 4060S) and AKT (#4685) were from Cell Signalling Technologies; c‐MYC (sc40) and BCL‐2 (sc‐7382) were from Santa Cruz; VINCULIN (SAB4200080); JQ1 and venetoclax inhibitors were from Selleckchem.

### 2.7 Statistical analysis

Two‐sided Student's *t* test or two‐way ANOVA with Bonferroni post‐test were calculated using GraphPad Prism v5.0d (GraphPad Software). *P*‐values < .05 were considered statistically significant. *P < .05; **P < .01; ***P < .001. All mean values (±SD) are from 3 independent experiments.

## 3 RESULTS

### 3.1 Treatment with JQ1 inhibits growth and survival in CLL cell lines

**Figure 1:**
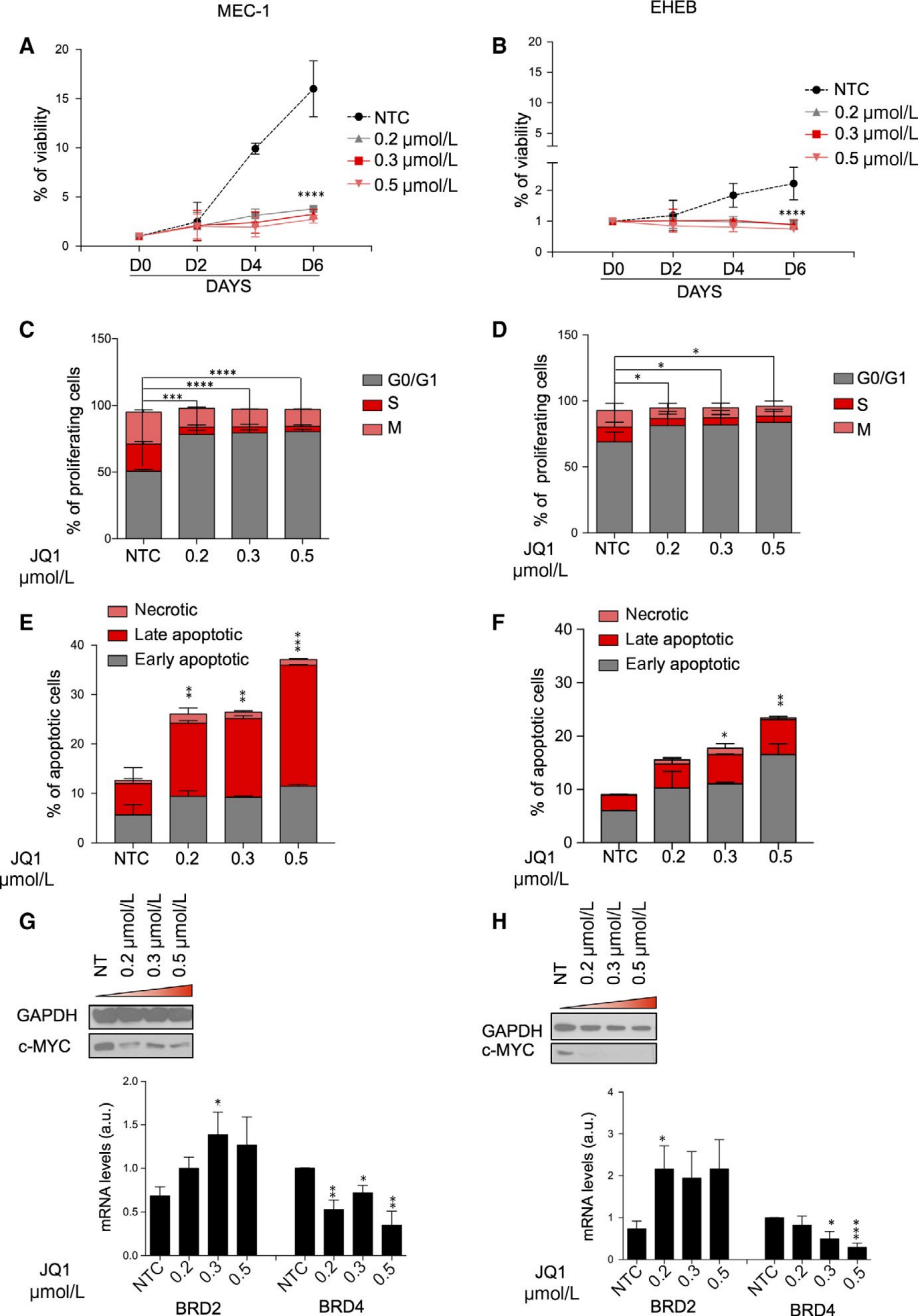
Treatment with JQ1 inhibits growth and survival in CLL cell lines. A, B, MEC‐1 and EHEB cells were cultured in the presence or absence of JQ1 and cell counts were measured every 48 h for 144 h. C, D, Cell‐cycle status of MEC‐1 and EHEB cells following 48 h of treatment with JQ1, as indicated. E, F, MEC‐1 and EHEB cells were treated with JQ1, as indicated, for 48 h. The percent of apoptosis was determined by staining with Annexin V/PI and flow cytometry. Columns represent the mean apoptosis of 3 independent experiments. G, H, upper panel: Western immunoblot of MEC‐1 and EHEB cells treated with the indicated concentrations of JQ1 for 48 h. Immunoblot analyses were conducted for the expression levels of c‐MYC; lower panel: real‐time PCR analysis of BRD2 and BRD4 mRNA levels in MEC‐1 and EHEB cell lines treated for 48 h with different JQ1 concentrations, as indicated

We first determined the JQ1 effect on the growth and survival of both in MEC‐1 and EHEB CLL cell lines. JQ1 treatment was associated with marked reduction in cellular viability (Figure 1A,B) and increased the percentage of G1‐phase of the cell cycle (Figure 1C,D). Treatment with JQ1 induced dose‐dependently apoptosis of CLL cells (Figure 1E,F and Figure S1A). Western analysis of the protein lysates showed that treatment with JQ1 reduced the protein expressions of c‐MYC protein (Figure 1G,H, upper panel) in MEC‐1 and EHEB cell lines. Accordingly, the qPCR analysis showed that JQ1 treatment attenuated the mRNA expression of BRD4 (Figure 1G,H, lower panel), which is a direct regulator of c‐myc expression. All together these data are in line with most of the observations with other cancers and suggest that BET inhibitors could be exploited also in the CLL context.

### 3.2 Combined treatment with JQ1 and venetoclax exerts synergistic lethal activities against CLL cell lines

**Figure 2:**
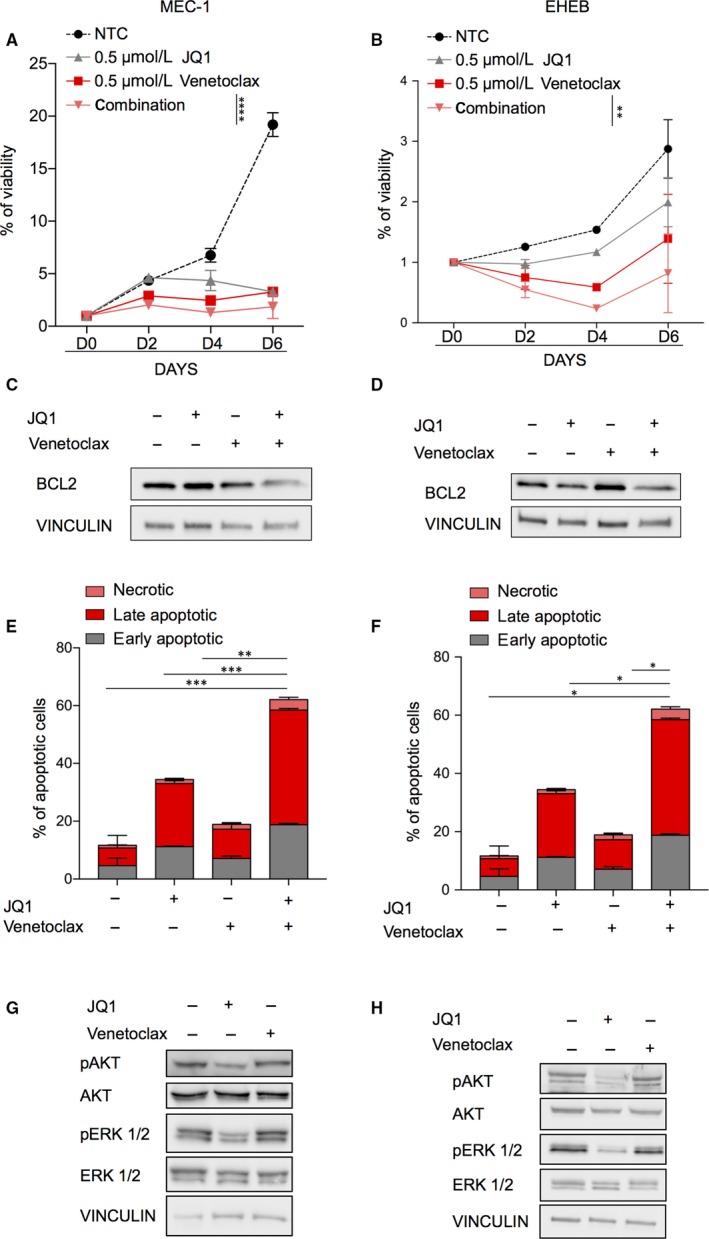
Combined treatment with JQ1 and venetoclax exerts synergistic lethal activities against CLL cell lines. A, B, MEC‐1 (left) and EHEB (right) cells were cultured in the presence or absence of JQ1 and venetoclax at indicated concentration. Cell counts were measured every 48 h for 144 h by CTG assay. C, D, Bcl2 protein expression MEC‐1 (C) and EHEB (D) upon treatment with venetoclax, JQ1 and the association. E, F, MEC‐1 (left) and EHEB (right) cells were treated with JQ1 and venetoclax for 48 h. The percent of Annexin V/PI‐positive apoptotic cells was determined by flow cytometry. Columns, mean of 3 independent experiments. G, H, Representative immunoblots of MEC‐1 and EHEB cells treated with the JQ1 for 48 h. Immunoblot analyses were conducted for the expression levels of p‐ERK1/2, ERK1/2, p‐AKT, AKT and vinculin in the cell lysates

**Figure 3:**
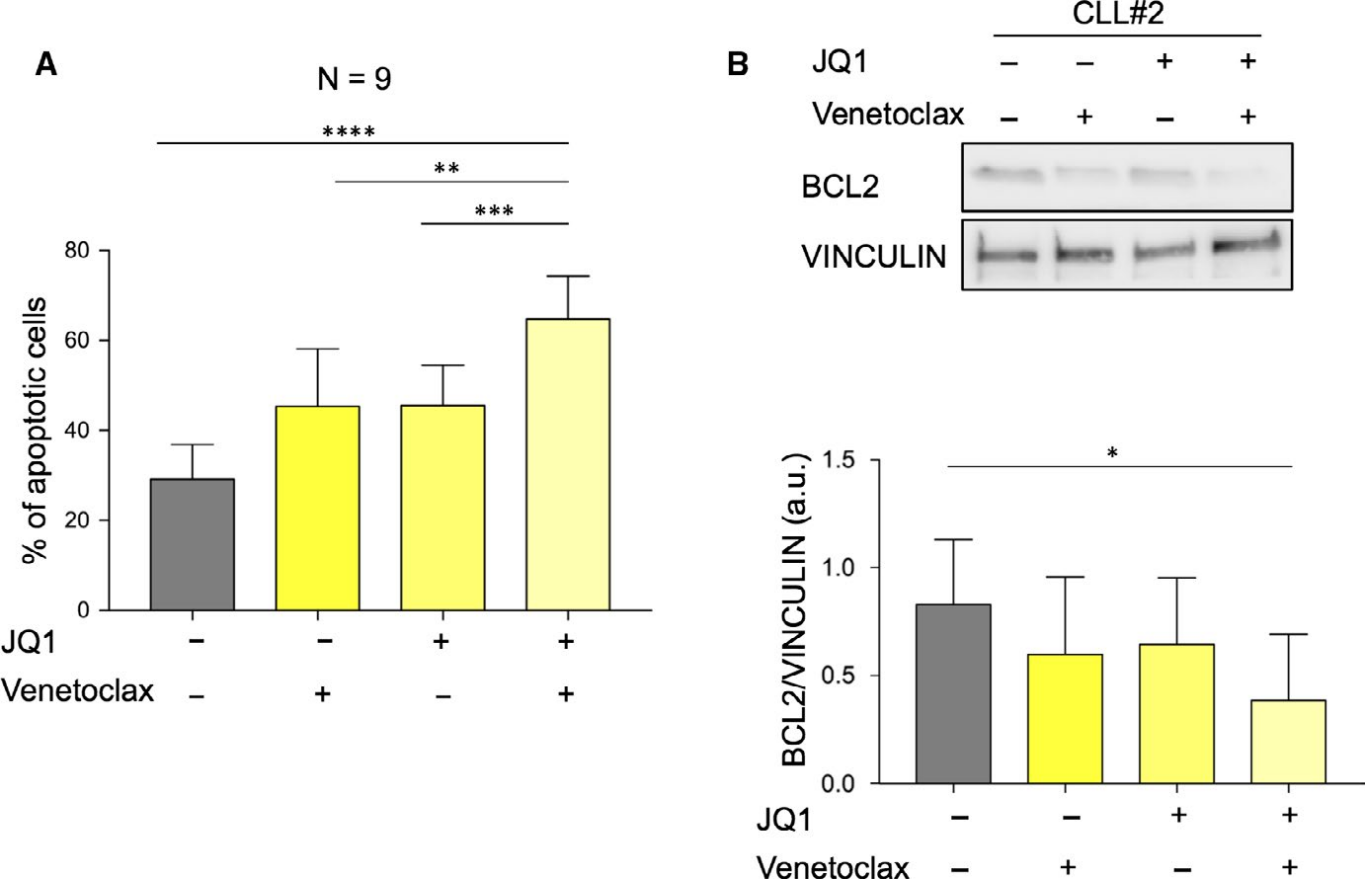
Co‐treatment with JQ1 and venetoclax affects the viability of ex vivo human CLL cells. A, Primary CLL cells were treated with JQ1 1 µmol/L, 0.5 µmol/L venetoclax alone or in combination for 72 h. The percent of non‐viable cells was determined by Annexin V staining and flow cytometry. B, upper panel: representative immunoblots of primary CLL cells treated with the indicated inhibitors for 72 h. Immunoblot analyses were conducted for the expression levels of BCL2 and vinculin in the cell lysates; lower panel: quantification of all analysed patients

To define whether pharmacological inhibition of BRD4 synergies with venetoclax regimen, we treated both TP‐p53‐mutated‐MEC1 and TP‐p53 wild‐type‐EHEB cells with a combination of venetoclax plus JQ1, observing a significantly higher reduction in cell viability compared to single treatments (Figure 2A,B). Moreover, due to the ability of JQ1 to modulate BCL2 expression,^22^ we observed that the association with venetoclax plus JQ1 favours a profound BCL2 downmodulation (Figure 3C,D) which may be the cause of such a remarkable apoptosis induction (Figure 2E,F and Figure S2A). Notably, treatment with JQ1 and venetoclax in combination synergistically induced apoptosis of CLL cell lines, with combination index (CI) <1.0 (Figure S2B). To recapitulate the mechanism of this synergy, it is worth to note that in some previously published works, it was showed that EGFR and insulin receptor families are direct targets of BRD4 in several cancer models. This strongly suggests that, by blocking BRD4, BET inhibition can regulate the PI3K signal in cancer cells.^23^ However, in xenograft models of endometrial cancer, JQ1 significantly increases the expression of PTEN, favouring the block of the PI3K/AKT signalling pathway.^24^ Very recently it was demonstrated that BRD4 inhibition induces apoptosis and growth arrest in glioblastoma cells via VEGF/PI3K/AKT regulation.^25^ Furthermore, JQ1 is also effective to reduce the levels of p‐Erk 1/2 in mouse models of pancreatic cancer and in anaplastic thyroid cancer cells where is often over‐activated. Similar results were obtained also in ovarian cancer cells.^26^, ^27^, ^28^

Accordingly, Western blot analysis showed that only JQ1 and not venetoclax reduced phosphorylation of ERK1/2 and AKT, potentially explaining the synergy of drugs in impairing CLL survival from a regulatory point of view (Figure 2G,H).

Altogether, these data indicate that treatment of CLL cells with BETi enhances the susceptibility to venetoclax.

### 3.3 Co‐treatment with JQ1 and venetoclax affects the viability of ex vivo human CLL cells

To assess whether the combinatorial therapy may affect primary CD19 positive lymphocytes from CLL patients, we treated freshly isolated CD19 positive CLL cells with JQ1, and venetoclax alone or the combination of the two drugs. Supplementary Table [Link] resumes the main features of CLL patients used in ex vivo experiments. Consistent with our previous observation, the combination is associated with a marked apoptosis induction and a pronounced reduction in BCL2 (Figure 3A,B and Figure S3A). These findings suggest that, as compared with treatment with each agent alone, co‐treatment with JQ1 and venetoclax markedly induced apoptosis and could prevent the development of resistant clones.

### 3.4 JQ1 treatment is effective against venetoclax‐resistant CLL cells

**Figure 4:**
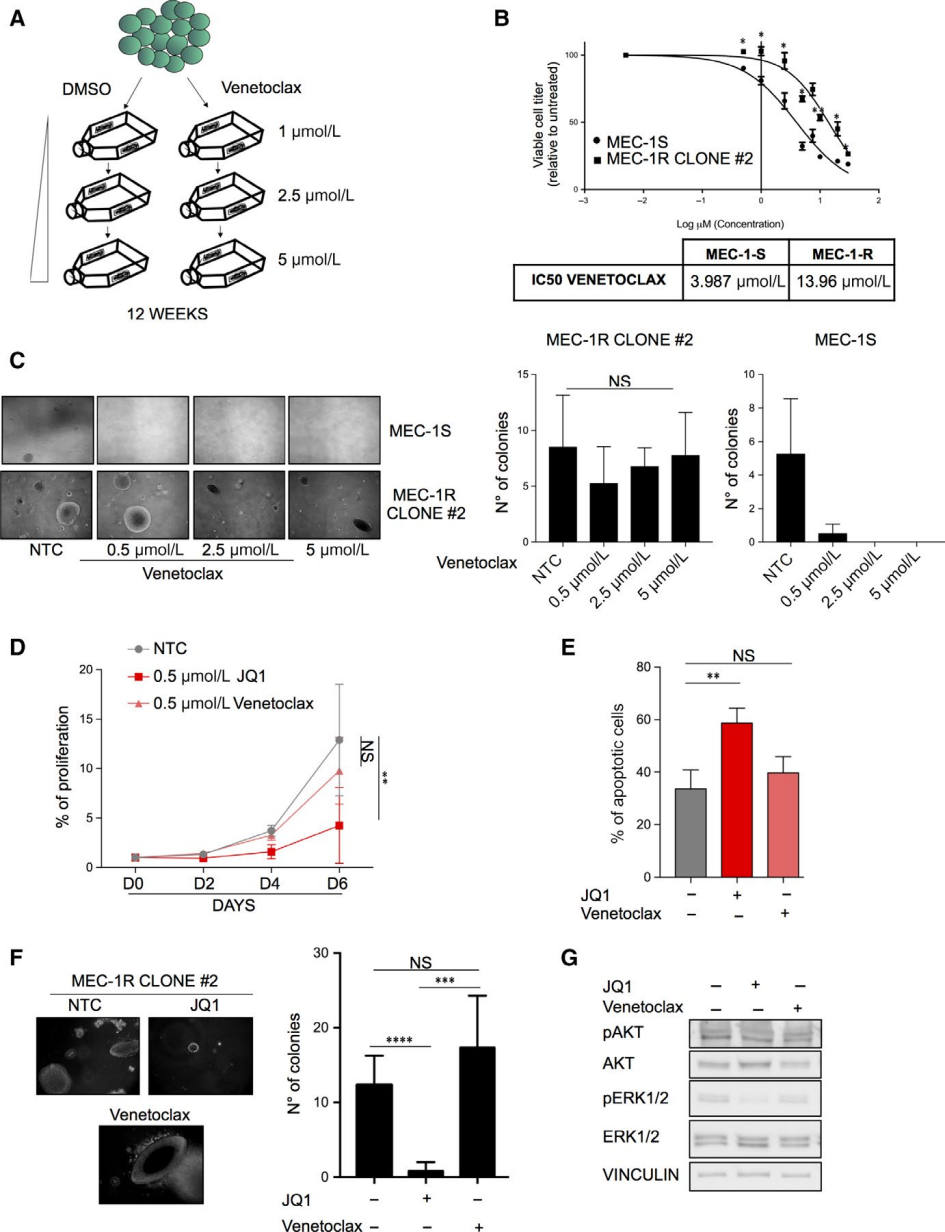
JQ1 treatment is effective against venetoclax‐resistant CLL cells. A, Schematic representation of venetoclax‐resistant MEC‐1 clones generation. B, Dose‐response curves of MEC‐1 cells lines treated for 48 h with JQ1 and analysed by CTG assay; IC‐50 values of venetoclax in MEC‐1 sensitive and resistant cells. C, Left panel: representative images of soft‐agar growth assay in MEC‐1 cells treated with different venetoclax concentrations (0.005‐0.5‐2.5‐5 µmol/L) for 11 d and quantification of soft agar colony formation; right panel: quantification of soft agar colony formation in MEC‐1 cells treated as previously described. D, Cells were cultured in the presence or absence of JQ1 and cell counts were measured every 48 h for 144 h by CTG assay. E, venetoclax resistant cells were treated with JQ1 72 h. The percent of apoptotic cells was determined by Annexin V staining and flow cytometry. F, Representative images and quantification of soft agar colony formation of venetoclax‐resistant cells after 15 d of treatment with 0.5 µmol/L venetoclax and 0.5 µmol/L JQ1. G, Representative immunoblots of MEC‐1 and EHEB cells treated with the JQ1 for 48 h. Immunoblot analyses were conducted for the expression levels of p‐ERK1/2, ERK1/2, p‐AKT, AKT and vinculin in the cell lysates

Lastly, we investigated the efficacy of JQ1 in venetoclax‐resistant clones. We established MEC‐1 resistant cells to venetoclax by culturing MEC‐1 cells in the continuous presence of escalating doses of venetoclax (Figure 4A). MEC‐1 resistant cells exhibited significantly higher IC50 values for venetoclax (MEC‐1:3.987 µmol/L vs MEC‐1R: 13.96 µmol/L; Figure 4B). Colony formation assay further supports the resistance of MEC‐1 cells to venetoclax (Figure 4C).

Importantly, JQ1 treatment inhibited proliferation (Figure 4D), significantly increased cell death (Figure 4E) and abrogated colony formation (Figure 4F). Finally, Western analysis showed that JQ1 abrogate phosphorylation of ERK1/2 and AKT, suggesting that the anti‐tumour effect of BRD inhibition in venetoclax‐resistant cells could depend on an impairment of this protein activity (Figure 4G). Lastly, by treating primary CD19 cells collected from a patient with venetoclax resistant disease, the apoptotic response is slightly increased (Figure S4A,B). Overall, these data suggest that BRD inhibition could be an intriguing second‐line therapy regimen in CLL, in the case of emergence of venetoclax resistance, but further investigations are needed.

## 4 DISCUSSION

Dissecting the process of resistance to targeted therapies is important to define new therapeutic strategies. Our data demonstrated that (a) the BET inhibitor JQ1 has an anti‐tumour effect in CLL; (b) the combinatorial treatment of venetoclax plus JQ1 highly improves the apoptotic effect of single treatments, both in cell lines and patient‐derived models; (c) CLL cell line MEC‐1 can easily develop resistance to venetoclax; (d) JQ1 is strongly efficacious as second‐line strategy, in the case of emergence of venetoclax‐resistant clones.

Overall, these data raise some concerns on the utility of single agents' schedules with new drugs in CLL, and potentially in other cancers. While chemotherapy was historically designed as a poly‐chemotherapy, where various agents target different mechanisms of cancer maintenance, new drugs were referred to the paradigm of the precision medicine due to high selectivity and precision.

While selectivity may be a hallmark of drug safety, the innate genetic and epigenetic heterogeneity of cancer suggest that different biological mechanisms cooperate to sustain human cancers and therefore a single‐drug approach is insufficient to eradicate them.

Clinical data, biological experiments and our data have indeed demonstrated that the single administration of these drugs is far away from the dream to eradicate CLL, or, better to cure CLL. Our data support the rational of combinatorial strategies to be administrated up‐front the development of resistances. Conversely, whether resistant clones are already emerged, second‐line treatment with targeted agents can still be worth. We believe that venetoclax plus BET inhibitors may represent a highly effective strategy to eradicate CLL. In particular, combinatorial therapy venetoclax plus Beti should be considered as a challenging approach to treat those patients with a highly aggressive form of CLL, those with mutant TP53, or to enhance the efficacy of venetoclax‐base therapy in those patients where responses are partial. Lastly, it should be also considered that therapies with venetoclax, as with ibrutinib and idelalisib, appear to last indefinitely. An intensification schedule may eventually offer the change to limit the duration of the therapy allowing to eradicate the disease rather than controlling it.

## CONFLICT OF INTEREST

The authors confirm that there are no conflicts of interest.

## AUTHOR CONTRIBUTIONS

GC and AM designed experiments; PN, PC, GP and VG provided human samples; GC, MFL and BM performed experiments; AM, GC and MFL wrote the manuscript; GC, MFL, MB, AG, GS, RT, AC and AM review the data.

## Data Availability

The data that support the findings of this study are available from the corresponding author upon reasonable request.
