# Supplementary material for: Inhibition of bromodomain and extra‐terminal proteins increases sensitivity to venetoclax in chronic lymphocytic leukaemia
Source: J Cell Mol Med. 2019 Dec 10;24(2):1650–7. doi: 10.1111/jcmm.14857 (PMC6991693; doi:10.1111/jcmm.14857)
Supplement: Supplementary file 1 [file JCMM-24-1650-s001.pdf]

SUPPLEMENTARY FIGURES

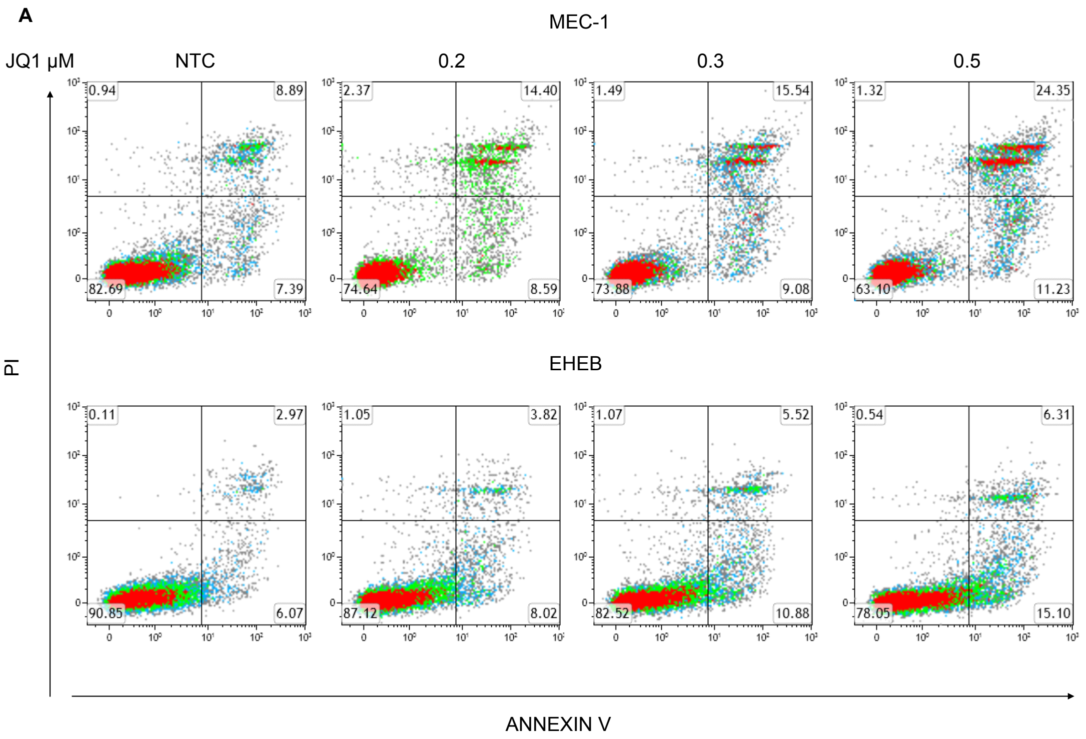

Supplementary Figure S1: Cytometric analysis of MEC-1 and EHEB cell lines were treated with JQ1

(A) Dot plot of flow cytometric analysis of MEC-1 and EHEB cell lines treated with the indicated concentrations of JQ1 for 48 hours.

A

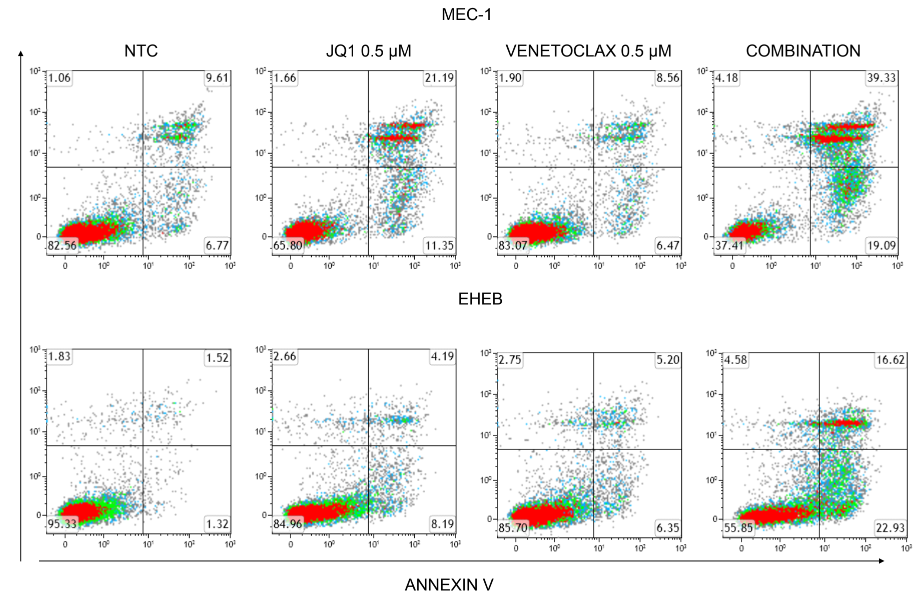

B

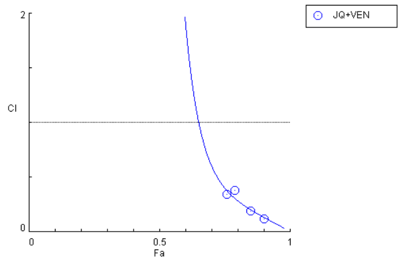

Combination Index

Total Dose	Fa	CI Value
1.0	0.76	0.34726
1.5	0.85	0.18903
1.5	0.79	0.37720
2.0	0.9	0.12060

Supplementary Figure S2: combined treatment with JQ1 and ibrutinib exerts synergistic lethal activity in CLL cell lines

(A) Dot plot of flow cytometric analysis of MEC-1 and EHEB cell lines treated with the indicated concentrations of JQ and Venetoclax alone or in combination, for 48 hours. **(B)** CLL cells were treated with JQ1 and Venetoclax for 48 hours. The percent of nonviable cells was determined by CellTiter-Glo assay. Median dose effect and isobologram analyses were performed utilizing Calcu-Syn. CI values 1.0 indicates a synergistic interaction of the two agents in the combination.

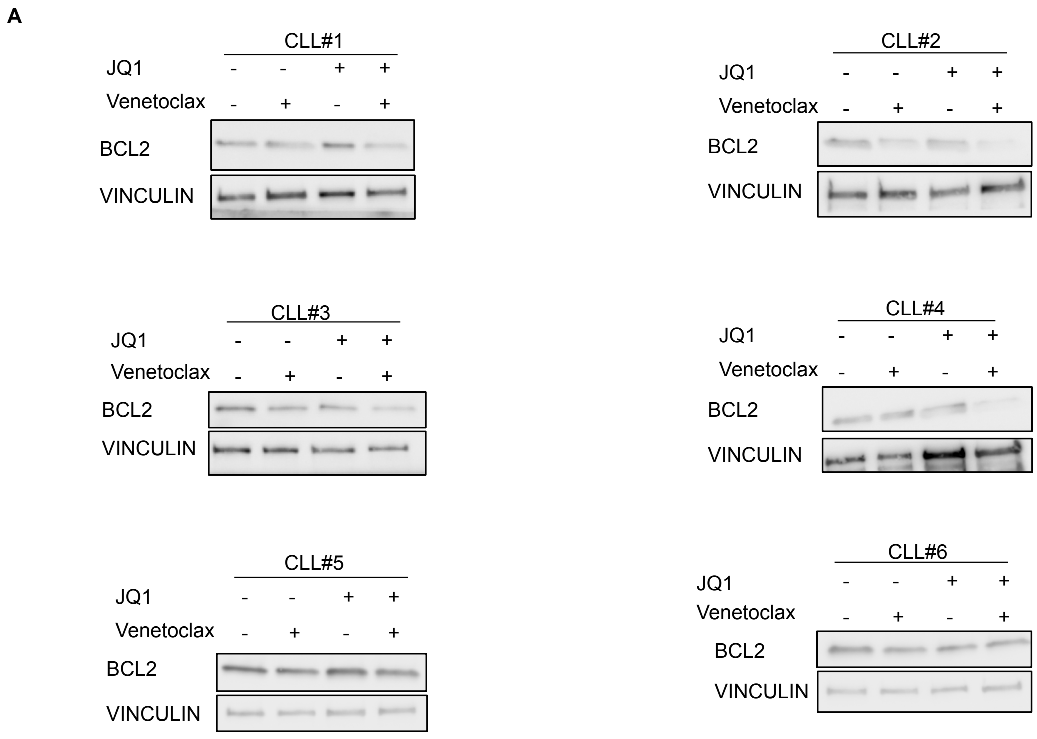

Supplementary Figure S3: Full Western Immunoblot of CLL patients

A) Western immunoblot of primary CLL cells treated with JQ1, Venetoclax and combination for 48 hours. Immunoblot analyses were conducted for the expression levels of BCL2.

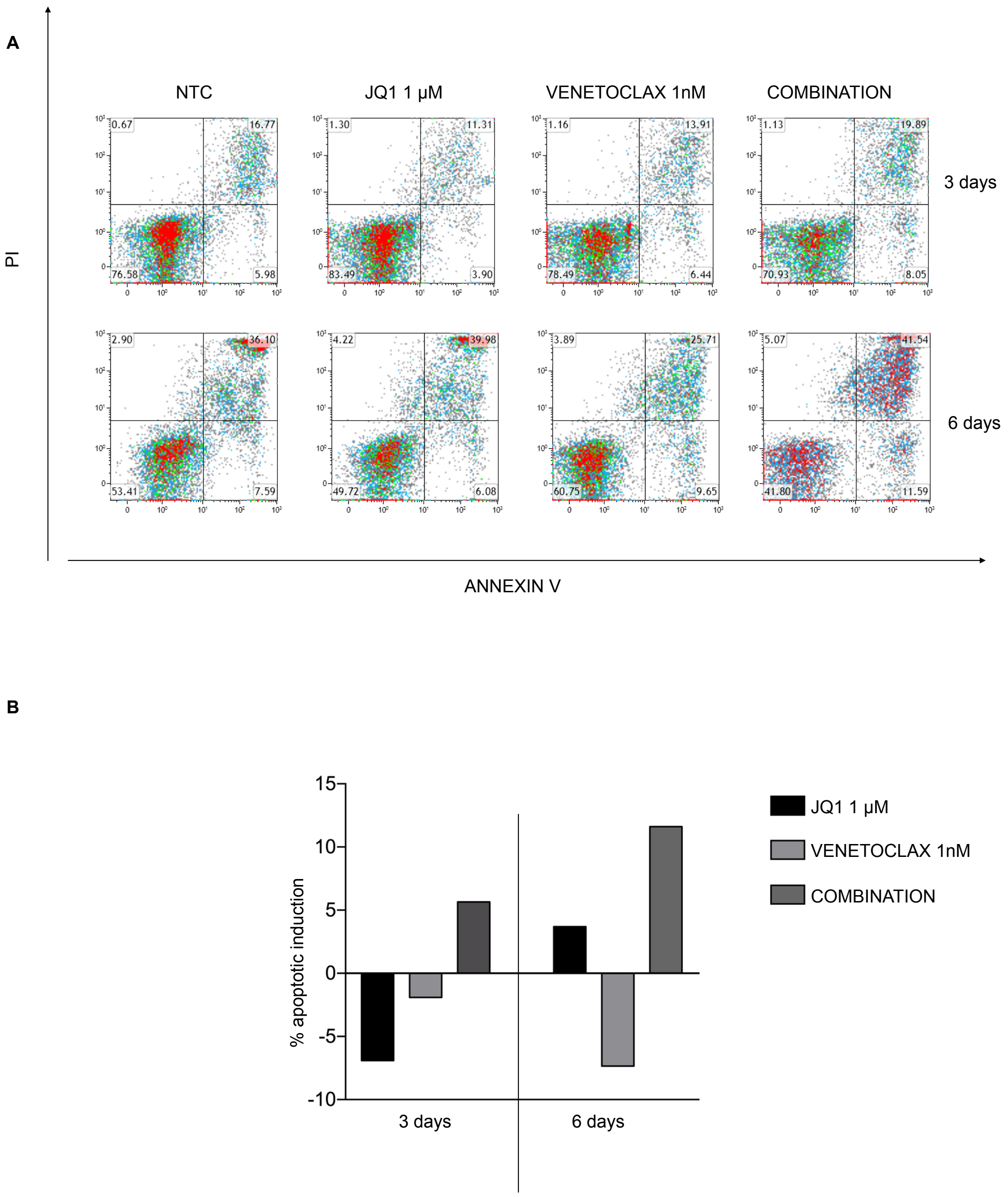

Supplementary Figure S4: JQ1 re-sensitizes Venetoclax-resistant patients to venetoclax

(A) Dot plot of flow cytometric analysis on the peripheral blood in Venetoclax-resistant patient cells. **(B)** Apoptosis-induction calculated on Venetoclax-resistant patient cells, treated with JQ1 and Venetoclax at indicated concentrations alone or in combination.

TABLE 1: Patients features

	Patient characteristic		Diagnosis					At the collection of the samples				Cytogenetics					Therapy		Follow up
code	age	sex	stage Rai	WBC	Hb	PLTS	adenophaty	WBC	Hb	PLTS	adenophaty	CD38/ZAP70	TP53 status	IGVH	cytog. (FISH)	trisomy 12	yes/no/WW	FCR/BTK…	
#1	79	M	0	17000	normal	normal	not significant	134800	13,5	127.000	spleen 17,5 cm, lfn ltc max 3 cm ltc, small inguinal lfn	At diagnosis, CD38 neg; in 2003, bimodal CD38; in 2005, CD38+, ZAP70+	NA at diagnosis. 2019 ongoing	unmut	del13q14.3	no	ww until 2005; 2005: FC --> good PR, allergic reaction --> stop. 2008: R-OxDA. 2011: LDT<6 months, Benda. 2013: leukeran. 2014: oxDHA --> PR --> ibrutinib 2015, PR. 2018: PD for leukocytosis --> venetoclax from 2019.	ibrutinib 2015-2018. 2018: venetoclax	2019: MMR 0,002%
#2	71	F	1	49500	12,9	284.000	axillary max 40 mm, paratracheal 25 mm. Interaortocaval, crural, paraaortic, iliac, obturator max 25 mm	na	na	na	as diagnosis	CD38 neg, ZAP70 neg	NA	unmut	del17p, del13 at diagnosis. In 2011, also monosomy 17.	no	2010: oxDHA.2012: Epoch--> PD--> OxDHA. Worsening clinical conditions --> leukeran.	no	lost to fu, likely dead.
#3	74	F	1	6230	12	198.000	ltc 5-20 mm,axillary 30 mm,several abdominal 30mm, lomboaortic tissue 60 mm, iliac max 20 mm.	2180	11	147.000	during treatment (very good partial response)	CD38 neg, ZAP70 borderline	neg	NA	del11	no	bendamustin from 2017, then discovery of lung cancer -->stop after 4 cycles.	no	ongoing therapy for lung cancer. CLL still in very good partial remission
#4	70	F	0	15900	14,3	262.000	not significant	166000	13,1	247.000	Hepatic hylum: adenopatic mass 85 mm; lymphonodes above pancreas max 32 mm; common iliac sn max 24 mm, external iliac max 18 mm.	CD38 neg, ZAP70 neg	NA	3 riarrangements: 2 mutated, 1 unmutated	at diagnosis:normal	no	ww	no	2019: alive, leukocytosis slowly progressive (WBC 90.000), no anemia or plts reduction.
#5	79	F	0	15420	normal	normal	not significant	6720	13,9	140.000	2017: axillary max 40 mm, laterocervical max 26 mm	CD38pos, ZAP70 pos	wt	unmut	trisomy 12 at diagnosis. 2015: trisomy 12, del 11q (ATM). 2017: tri 12, del 11q, del 17p	yes	2010: progression (adenopathies and LDT< 6 months)--> R-Benda, RC. 2015: relapse, lymphocytosis --> R-benda, good PR. 2017: relapse, hyperleukocytosis and AiHA: R-CVP, then ibrutinib from 2017. Hyperleukocytosis and suspected Richter Sy. from 2019: OxDHA, brief response. --> Venetcolax from 2019	ibrutinib 2017 --> 2019. venetoclax from 2019	venetoclax just started
#6	68	M	0	24300	14,3	229.000	14mm ltc	6010	13,3	131.000	CR at the end of treatment	CD38 pos, ZAP70 border line	wt	1 mut clone and 1 unmut clone	wt	no	ww till 2017, when the patient was treated with 6 cycles of R-Bendamustine for adenopatic progression and thrombocytopenia (85.000) --> CR	no	CR
#7	81	F	1	24960	14,2	296.000	ltc max 17mm, axillary mas 18mm, inguinal max 30mm, hepatic hilum 36mm, interaorticaval 26mm	10290	13,3	292.000	periportal lfn 16 mm	CD38 neg, ZAP70 neg.	NA	unmut	del 11q (ATM) and del13q	no	ww Adenopatic progression--> 2016: R-ibrutinib (Gimema LLC 1114).	yes, ibrutinib from 2016. CR	CR in ibrutinib
#8	68	F	0	19120	14,1	244.000	not significant	18790	14,1	191.000	spleen 12 cm, not significant lymphadenopathies.	CD38 neg, ZAP70 neg.	mut	mut	del13 at diagnosis. 2015: p53 mut, del 17p 10%.	no	Hyperleukocytosis and anemia --> R-benda in 2013. PD in 2015; p53 mut, del17 10%--> ibrutinib 2015. good PR	yes, ibrutinib from 2015	penumonia in 2017--> ibrutinib suspension. CLL not requiring treatment (GB 49870, spleen 15 cm).
#9	73	M	0	10500	14,1	172.000	not significant	2730	12,6	80.000	sample taken during treatment; pre treatment, in October: diffuse adenopathies max 3 cm. Spleen 20 cm, epatomegaly	CD38 neg, ZAP70 neg.	wt	mut	NA (del17 neg,other exams not available)	no	ww 2002-2009. 2009: FCR for splenomegaly (20 cm and thombocytopenia 97.000). 2 cycles, then stop for pneumonia. PD in 2017 --> R-benda --> CR	no	CR
